# Structural violence and the uncertainty of viral undetectability for African, Caribbean and Black people living with HIV in Canada: an institutional ethnography

**DOI:** 10.1186/s12939-022-01792-4

**Published:** 2023-02-17

**Authors:** Apondi J. Odhiambo, Patricia O’Campo, La Ron E. Nelson, Lisa Forman, Daniel Grace

**Affiliations:** 1 Dalla Lana School of Public Health, University of Toronto, Toronto, Ontario Canada; 2 St, Michael’s Hospital, Li Ka Shing Knowledge Institute, Toronto, Canada; 3 Yale School of Nursing, New Haven, CT USA

**Keywords:** HIV, Retention in healthcare, Adherence to ART, Institutional ethnography, African, Caribbean, And black, Structural violence, Social determinants of health, Equity, Social justice

## Abstract

Biomedical advances in healthcare and antiretroviral treatment or therapy (ART) have transformed HIV/AIDS from a death sentence to a manageable chronic disease. Studies demonstrate that people living with HIV who adhere to antiretroviral therapy can achieve viral suppression or undetectability, which is fundamental for optimizing health outcomes, decreasing HIV-related mortality and morbidity, and preventing HIV transmission. African, Caribbean, and Black (ACB) communities in Canada remain structurally disadvantaged and bear a disproportionate burden of HIV despite biomedical advancements in HIV treatment and prevention. This institutional ethnography orients to the concept of ‘structural violence’ to illuminate how inequities shape the daily experiences of ACB people living with HIV across the HIV care cascade. We conducted textual analysis and in-depth interviews with ACB people living with HIV (*n* = 20) and health professionals including healthcare providers, social workers, frontline workers, and health policy actors (*n* = 15). Study findings produce a cumulative understanding that biomedical HIV discourses and practices ignore structural violence embedded in Canada’s social fabric, including legislation, policies and institutional practices that produce inequities and shape the social world of Black communities. Findings show that inequities in structural and social determinants of health such as food insecurity, financial and housing instability, homelessness, precarious immigration status, stigma, racial discrimination, anti-Black racism, criminalization of HIV non-disclosure, health systems barriers and privacy concerns intersect to constrain engagement and retention in HIV healthcare and ART adherence, contributing to the uncertainty of achieving and maintaining undetectability and violating their right to health. Biomedical discourses and practices, and inequities reduce Black people to a stigmatized, pathologized, and impoverished detectable viral underclass. Black people perceived as nonadherent to ART and maintain detectable viral loads are considered “bad” patients while privileged individuals who achieve undetectability are considered “good” patients. An effective response to ending HIV/AIDS requires implementing policies and institutional practices that address inequities in structural and social determinants of health among ACB people.

*“Let us first deal with putting everyone on treatment, ensuring people access medication consistently and people have proper housing, nutrition, mental healthcare and psychosocial package that deals with racism, the justice system, criminalization, immigration and all these other issues. There are so many other barriers before we [Black people] get there [undetectable levels], and we cannot leave anyone behind.” (Bob, male, living with HIV)*.

## Introduction

This study is part of a larger institutional ethnographic project that explored how social relations shape the daily experiences of African, Caribbean, and Black people living with HIV across the HIV healthcare and treatment cascade [1, 2]. Black communities are structurally disadvantaged and bear a disproportionate burden of HIV despite biomedical advances in HIV treatment and prevention in Canada. Although the ACB communities make up less than 3% of the Canadian population, they account for 30% of HIV prevalence and 15% of new HIV infections [3]. Several ground-breaking clinical trials have demonstrated that antiretroviral treatment (ART) can suppress the amount of HIV in the blood to levels that cannot be detected via standard tests [4] or a state of undetectability [4–8]. Undetectability is fundamental for optimizing health outcomes, decreasing HIV-related mortality and morbidity, and preventing onward transmission of the virus [4–6].

Achieving and maintaining an undetectable viral load for people aware of their HIV-positive status requires consistent ART adherence [9–12]. At the same time, adherence to ART requires people living with HIV to remain engaged in continuous care, have routine follow-up services to monitor treatment efficacy, and take medication without interruption and discontinuation following diagnosis and treatment initiation [13–15]. Studies have associated low retention in care and non-adherence to ART with suboptimal health outcomes and high mortality rates [9, 16–18]. Discontinuation of healthcare and treatment interruption can result in drug resistance, substantially limit medication options, and increase the viral load, increasing the likelihood of opportunistic infections, death, and risk of onward HIV transmission [5, 12].

Despite attentiveness to biomedical advancements and global initiatives aimed towards ending HIV, research focusing on the HIV care cascade has shown that inequities manifest through structural and social determinants of health (SDOH) [19–21] to negatively affect linkage, access, engagement and retention in HIV care, and adherence to ART across the care cascade [22]. While there is limited race-based data on engagement and retention in the HIV care cascade, extensive literature shows that inequities in structural and SDOH are markers of poor health outcomes and suffering for ACB people living with HIV in Canada [18, 23, 24]. Studies show that vulnerable and marginalized populations such as ACB people are sometimes lost to care compared to other communities due to inequities [1, 2, 18, 25–28]. We frame these inequities as products of a socio-political context characterized by structural violence and human rights violations [29].

### Structural violence, inequities, and structural violation of human rights

Structural violence is a concept for understanding the material harm, social injustices and vulnerabilities to increased risk for diseases, suffering, lack of access to care, and poor health outcomes caused by broader social, political, economic and legal forces that determine people’s social positioning and shape their social world [30]. Introduced by Johan Galtung (1969), structural violence is conceptualized as an assemblage of complex, invisible, indirect, and insidious processes embedded within institutions, laws, and policies that constrain human agency and cause harm to individuals or groups who hold limited or no power by denying or restricting access to the resources they need to survive while privileging others [31–33]. Farmer explains that structures produced and sustained by “hard surfaces of life” such as racism, unemployment, poverty, and political violence result in inequities in the distribution of power and resources, which in turn constrain agency and influence the ability to make decisions and fulfill essential needs necessary for overall health and wellbeing [34, 35]. Farmer indicates that structural inequities, whether in the labour market, the justice system, or in access to healthcare, quality housing, and healthy food, which often correlate with poverty, racism, disenfranchisement—are “embodied and experienced as violence” [36].

Health scholars define structural violence as “a host of offensives against human dignity, including extreme and relative poverty, social inequalities ranging from racism to gender inequality, and the more spectacular forms of violence that are uncontested human rights abuses” [29]. Structural violence manifests through the unequal distribution of power, unjust policies, and institutional practices that produce and reinforce structural inequities that restrict access to equitable determinants of health [37]. Farmer emphasizes that structural violence constitutes a violation of human rights when power distributed through structures constrains individual human agency to the extent that meeting basic human needs becomes unattainable [35, 37].

Medical anthropologists and sociologists have argued that epidemics such as HIV and related consequences result from structural processes, forces, and forms of violence that interface to shape and constrain the agency of individuals [38–40]. Farmer used the concept of structural violence in global health to demonstrate that social, economic and political forces such as racism, sexism and political violence shape the HIV epidemic and health outcome [41]. As such, Farmer calls for a reduction in “structural vulnerability” and poverty as an approach to eradicating HIV/AIDS.

Generally, investigation of structural oppression and violence is rare. Most of the health research in Canada and globally focuses on individuals. More specifically, individuals are seen as the source of problems. In HIV/AIDS response, the impact of structural violence on people’s ability to reach undetectability is rarely investigated because linkage, access, engagement, retention, and adherence to HIV healthcare and treatment are perceived as an individual’s responsibility. HIV discourses, research, and healthcare practices primarily focus on individuals’ knowledge about HIV, lifestyle risk factors, behaviours and actions, and acute clinical care while largely ignoring structural factors constraining health practices among Black people [23, 42–44]. Current Canadian HIV/AIDS prevention laws and policies primarily focus on screening, surveillance and policing of people living with HIV, particularly ACB immigrants while ignoring the SDOH and the disproportionate impact of HIV/AIDS on these communities [45, 46]. While not disregarding the significance of individual-level activities and clinical care, an emphasis on individual determinants obscures and ignores power relations and inequities that shape the social world of Black people. Mbuagbaw et al. (2020) critique the lack of literature on viral suppression in Black communities and call for research addressing the structural factors that impede the ability to reach and maintain undetectability.

This institutional ethnography (IE) study orients to the concept of structural violence to broaden the thinking and practices of HIV healthcare and treatment to establish a stronger relationship between individuals’ material conditions and disease, inequities, SDOH, and human rights [37]. The study explores how structural violence shape and constrain retention in HIV healthcare and adherence to treatment.

## Method

Developed by Dorothy E. Smith, IE is a “sociology for the people” [47]. The ontology of IE calls for research to start from the individuals’ social world, mapping their standpoints, knowledge, and concerns from everyday experiences to understand how institutional processes are coordinated [48–50]. Structural violence is a problem in the everyday social world that is not enacted by individuals working within institutions but by effects of “ruling relations” that shape and organize people’s day-to-day lives [51–53]. IE also pays attention to the concept of work, which Smith defines as “anything done by people that takes time and effort …It means much more than what is done on the job” [47]. In line with this description, IE allows researchers to question how individuals negotiate and engage in invisible work practices to reach a particular work goal. Furthermore, IE identifies and examines text that organizes institutional processes and discourses, legislation, and policies. Data collected from the lived experiences of Black communities and governing texts help locate disjunctures between discourses and practices of HIV healthcare and the actualities of Black communities, illuminating structural violence produced by ruling relations within and beyond Canada’s healthcare system. Therefore, this study uses IE to illuminate Black people’s everyday activities and makes visible how social structures and institutional processes coordinating Black people’s social world enact structural violence to constrain their “health work” of retention in care, adherence to ART, and achieving and maintaining undetectability, and as such violate their right to health [47, 52–55].

### Data collection

This article presents data collected between May 2019 and October 2020 from ACB people living with HIV (*n* = 20) and health workers (*n* = 15) involved in HIV healthcare delivery. Data collection began immediately upon receiving research ethics approval. All participants reviewed study information and provided informed consent to participate in the study. In some instances, pseudonyms were used to protect Black participants’ privacy and professional roles to reference health workers.

The study began by exploring the experiences of ACB people involved in the institutional practice of HIV healthcare. Black participants were recruited through community-based agencies and healthcare centres and purposive sampling. They were included if they self-identified as a person living with HIV and African, Caribbean, or Black. Using semi-structured interview guides, ACB participants were asked about their work of engaging in HIV healthcare and adherence to ART, their challenges, and the steps they took to navigate the obstacles. The participants were probed further about their everyday experiences to understand how broader structural factors beyond their control shaped their health work of adhering to ART. Interviews with key informants lasted 60–120 minutes.

The study also included participants located in various social positions because IE’s interest is in going “beyond the interchanges of frontline settings to track the macro institutional policies and practices that organize those local settings” [56]. Consequently, purposive sampling methods was used to identify and recruit health workers. Health workers recruited included healthcare providers contracted to work in public healthcare hospitals and community healthcare centres, pharmacists, social workers, immigration panel physicians and frontline workers based in local AIDS service community organizations. Using a separate semi-structured interview guide, health workers were asked about their everyday experiences of delivering healthcare or social support to ACB people living with HIV. The questions focused on the challenges ACB people face trying to engage and stay in care and reach undetectability. Interviews with health workers ranged between approximately 30 and 45 minutes. Texts were iteratively identified and analyzed, including legislative frameworks, policies and clinical guidelines, which participants referenced during the interviews [57–59]. Furthermore, within interviews, participants explicated how texts co-ordinated or mediated their work practices.

### Data analysis

The research process was an iterative process that occurred concurrently as the first author moved back and forth between data collection and analysis. Interviews were transcribed verbatim. The analysis started in the lived experiences of ACB people living with HIV. The focus was on understanding participants’ social worlds, including their stories, beliefs, knowledge, and perceptions about the work of adherence to ART and achieving undetectability through the reading of transcripts and listening to participants’ accounts [48, 60]. Textual analysis involved “processing interchanges” through reading, interpreting, and writing to produce “truth” about how institutions’ daily activities are coordinated [48, 49, 53]. The participants’ actual accounts were indexed to keep the analysis grounded on their experiences [61]. Transcripts and texts were systematically indexed, identifying emerging themes using NVIVO [62].

The concept of work was used as an analytical lens to organize the data around Black people’s and healthcare workers’ accounts of ‘health work activities. Furthermore, the analysis moved beyond personal experiences to discover how extended social relations and the social organization of HIV healthcare work mediate daily activities in the era of undetectability. Texts and other discursive materials directly stated or implied in the participants’ accounts as organizing their health work practices under every index heading were documented. A search of possible connections between Black people’s and healthcare workers’ experiences, health work knowledge, and the identified texts and discursive materials was done as part of indexing. Once indexing was complete, the indexes were synthesized and reorganized into themes. Rooting the analysis on the standpoints of ACB people, themes were classified based on institutional processes giving significant priority to structures, systems, and discourses that perpetuated structural violence and constrained retention in care and adherence to ART and what they did to respond to the challenges [63].

## Results

**Fig. 1:**
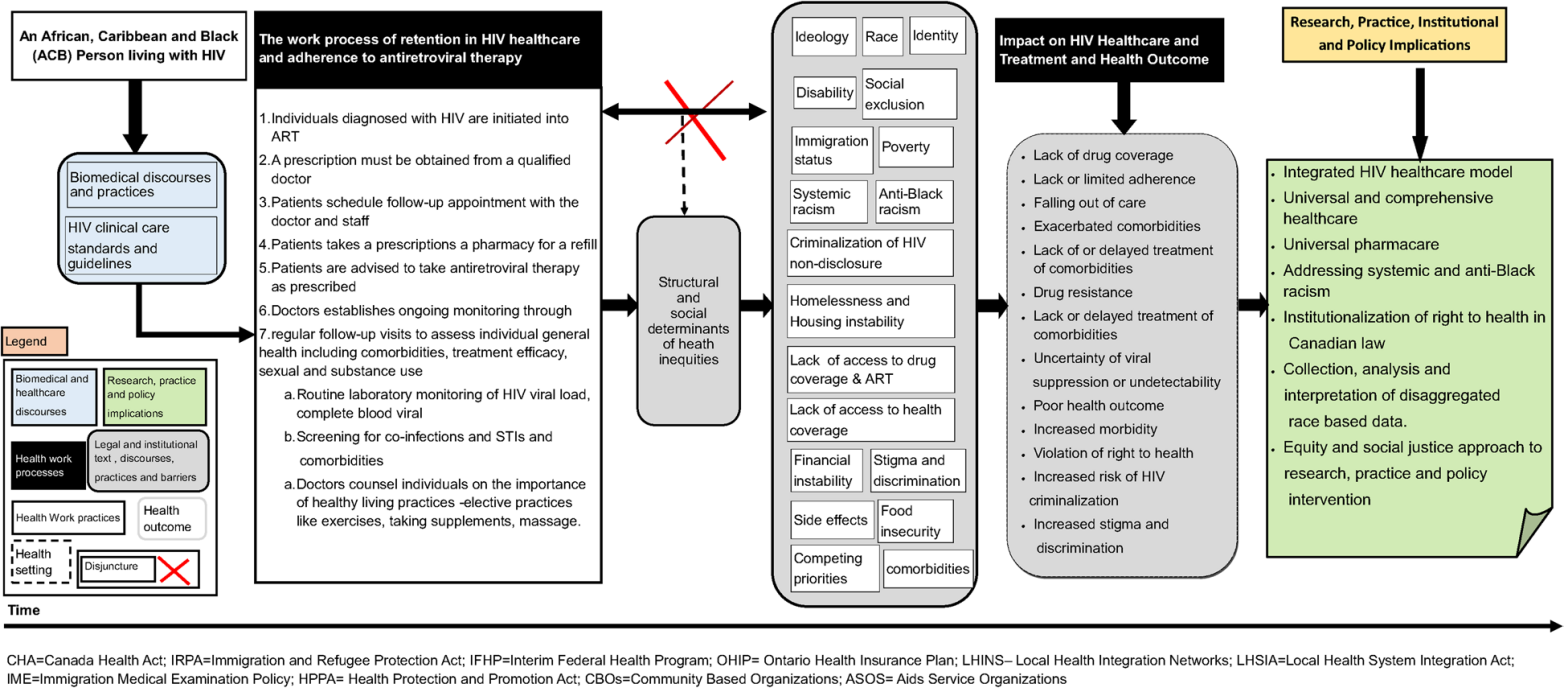
Map of the health work process of retention in HIV healthcare and adherence to Treatment for ACB people living with HIV in Canada

Black participants described how their knowledge and experiences of living with HIV existed in relationship to discourses about adherence to ART and undetectability. Mapping the experiences of ACB people, we found that ACB participants engaged in the discourses and the health work practices of HIV healthcare and treatment to achieve undetectable viral load and optimal health outcome. In this article, we refer to this work as “the health work process of retention in HIV healthcare and adherence to treatment” (Fig. 1). We first present the health work practices that participants engaged in that were reminiscent of the dominant discourses of HIV healthcare and treatment. We then show the disjuncture between Black people’s daily experiences and what HIV healthcare guidelines and practices count as the work of reaching and maintaining undetectability, driven by discourses of retention in care and adherence to ART. The concept of disjuncture refers to “moments when people know something from experience but are told or taught something quite different” [64]. By making explicit this disjuncture, we uncover that structural violence embedded within legislative frameworks, policies, and institutional practices produce structural and social determinants of health inequities that shape the social world of ACB people and constrain their health work of retention and adherence to HIV healthcare and ART. We then connect and show the ways that these inequities impact ACB participants’ health work process of retention in HIV health and adherence to ART. Lastly, we outline research, practice, institutional and policy implications of these findings.

### Mapping the work of adherence to ART and Achieving and maintaining Undetectability

Findings revealed that the health work of reaching undetectability involves a set of medical activities that require people living with HIV who are on ART to stay engaged in care and adhere to medication [57–59]. A critical aspect of this process is regularly scheduling medical appointments, attending clinical care and routine follow-up visits, monitoring HIV viral load and health outcomes through regular diagnostic laboratory work, and filling and refilling prescriptions. Adherence to ART also entails taking medication every day as prescribed. Participants explained that their physicians and pharmacists repeatedly stressed the importance of adherence to ART, highlighting possible consequences for failing to do so, including having drug resistance, a detectable viral load, and poor health. For example, Claude, who had detectable viral load when he first accessed HIV healthcare, explained that: “Rather than do the test [viral load monitoring] every six months, [the doctor] did them every three months to just observe that I am undetectable, and he talked about compliance. [The doctors] stressed that if I fail to take [my] medication, [I am] going to feel sick.”

Healthcare providers engaged in HIV healthcare explained that engaging in routine medical care was necessary to assess and monitor treatment efficacy and effectiveness, viral load levels, side effects, patient compliance, resistance, disease progression, and health outcomes. While several healthcare providers acknowledged their role in HIV healthcare delivery, they conceptualized the work of achieving and maintaining undetectability and optimal health outcomes as individual responsibility and accountability. Healthcare providers emphasized that people living with HIV are expected to take responsibility and be accountable for their health by continuously engaging in healthcare, adhering to ART, and following clinical advice. A physician based at an HIV clinic elaborated that:

There are things that I have no control over sometimes, like people not showing up for appointments. Today I just had one of those hour-long counselling sessions, but he ultimately did not show up, a young Caribbean man. Sometimes I can only do as much as I can. However, the patient also must do things I ask them to do, mainly instrumental things like, “here is the thing that you need to do for me to help you.”

### Locating the disjuncture: contesting the dominant discourses and work practices of HIV healthcare

Black participants living with HIV contested the discourse and practices of HIV healthcare and the concept of undetectability based on the various forms of oppression and inequities shaping their day-to-day realities. Although participants acknowledged the significance of HIV care and were happy with the availability of ART, many participants expressed that achieving and maintaining undetectable viral load was “more than taking a pill.” James, an immigrant who had lived in Canada for less than one year, expressed that: “undetectability is a fallacy in itself. [It] is not for Black bodies.” Participants described undetectability as a concept that is produced by standardized dominant discourses and institutional bodies that only privilege White people who can consistently engage in care, adhere to ART, and achieve viral suppression while ignoring the lived realities of Black people. Black participants explained that experiences of precarious immigration status, housing, employment, and lack of health insurance and drug coverage contributed to the uncertainty of reaching undetectability. Eunice elaborated that:

It [undetectability] is for White people who have the privilege of proper social support, housing and other benefits that come with them. We [Black people] struggle with so many other issues that being undetectable is almost a dream. You could be undetectable today and tomorrow lose your immigration case, interim coverage, medication [drug coverage], house or job because you rely on a work permit that is dependent on your legal status. Therefore, if you are undetectable, it is because you have the privilege of having a job. However, someone in the shelter system would probably never get undetectable until they can stabilize themselves and get their needs allowance.

Black participants expressed that biomedical discourses of HIV healthcare including the concept of undetectability produce class and racial differences by categorizing people living with HIV as “good” versus “bad” patients. People living with HIV who adhere to ART and manage to get their viral load to undetectable levels are labelled “good” patients. However, those perceived as nonadherent to ART and maintain detectable viral load are considered “bad” patients. Julian, a frontline worker who was living with HIV, expressed:

I think it [undetectability] puts people into the category of good or bad patients. The bad patient is the detectable one. I have seen patients who take the treatment well and everything, but they struggle to get even [achieve and maintain undetectable viral load]. They keep fluctuating. I do not know whether it is their body, but that does not make them bad clients because they do their best.

Black participants noted that the UNAIDS 90–90-90 treatment targets and the 95–95-97 Fast-Track strategy are far from being reached because of the systemic inequities faced by Black communities. Black people are disproportionately represented among the remaining 10–10-10%, leaving them behind. Lilian expressed: “We have seen already with the 90-90-90. But there are those 10% that are not being achieved, and it is even more than 10% because there are other things that prevent people from continuing to stay in care.” Participants asserted that conversations about undetectability should be grounded in the actualities of Black people. Additionally, tackling systemic barriers Black people face should be prioritized and integrated into HIV healthcare frameworks and programs.

### Uncertainty of achieving Undetectability: unpacking structural violence shaping health work of retention in care and adherence to ART

Black people’s description of their social world and lived experiences illuminated how structural violence embedded within health systems, legislative frameworks, and institutional practices produced inequities that constrain their retention in care, adherence to ART, and contribute to the uncertainty of achieving and maintaining undetectability. Structural inequities in relation to social determinants include a) financial instability, b) immigration status, homelessness and housing instability, c) food insecurity, d) health systems barriers, e) stigma and discrimination, f) systemic and anti-Black racism, g) comorbidities and h) criminalization of HIV non-disclosure.

#### Health system barriers and the work of managing HIV and related comorbidities

In addition to HIV, Black participants reported that they lived with one or more comorbidities such as ulcers, diabetes, high blood pressure, high cholesterol level, and mental health issues, including depression, anxiety, stress, and trauma. Participants associated their experiences of mental health with history of trauma from political conflicts and colonialism in home countries, social situations such as substance use and addiction, and the challenges and violence they face navigating discriminatory systems including housing, employment, immigration, healthcare, and welfare. Key informants and healthcare providers reported that health system barriers such as the lack of comprehensive healthcare and drug coverage and culturally responsive services, patient-provider power dynamics and inadequate time constrain Black people’s efforts to manage comorbidities and effectively achieve optimal health. Provincial public health insurance plan does not provide comprehensive coverage for all healthcare services.

When asked to reflect on healthcare delivery, Black people living with HIV emphasized the importance of “time”. There was expectation that healthcare providers would allocate adequate time to consult about Black patients’ overall health and subjective realities. However, Black people reported receiving suboptimal clinical care because the time allocated for consultation is often too short to adequately address their healthcare needs. The average time participants reported spending with their doctors was between 15 and 20 minutes. Francis explained that the physician was often in a hurry to “get him out of the door,” even after waiting for a long time before seeing the doctor. Despite experiencing multiple health issues or concerns, participants expressed that they were subjected to “one issue per visit” by their healthcare providers. Eunice expressed:

Time is an issue because you might have many questions and many things you want to discuss with your doctor. However, the doctor’s rule is to bring one issue at a time, do not bring many issues. If I do not talk about all the other issues affecting me, then the doctor cannot pick out the cause of my condition. If they have more time with you to explore and listen to your narrative, they should be able to pick up [that] this headache is not just a migraine, there is something [else]. The person could be stressed because the child is not doing well in school or following up on their refugee papers. It might not have anything to do with HIV.

Healthcare providers reported that they see so many patients that they find it challenging allocating patients the time they need to talk about their health issues. A social worker stationed at an HIV clinic noted that the short clinical time allocated “is very challenging, and sometimes patients seep through the crack.” Under the Ontario Insurance Plan (OHIP) schedule of Benefits and Fees, ‘fee for service’ is the model that doctors bill and generate revenue for their services. While physicians can address as many issues as they want, the ‘fee for service’ model permits them to bill only one issue per patient per visit.

Black participants living with HIV (*n* = 17) who primarily identified as recent immigrants to Canada indicated that lack of knowledge of how the Canadian healthcare system is organized obstructed their ability to navigate and access HIV-related healthcare services. Participants failed to look for general practitioners to treat their underlying conditions because of lack of knowledge of the roles played by different categories of healthcare professionals. Participants with HIV specialists as their sole healthcare provider expressed that they did not receive comprehensive care, including treatment for underlying health conditions. They noted that HIV specialists lacked adequate knowledge and willingness to address other health conditions. The HIV specialists prioritize monitoring viral loads and administering ART over assessing other underlying conditions and factors impacting Black people’s health. Further, many healthcare providers lacked cultural competency and interest to address all the health needs of Black people. The power imbalance between Black people and their doctors prevented them from self-advocating for more consultation time and culturally responsive care. Brenda, who was volunteering at a community healthcare centre and self-identified as a Black immigrant living with HIV, elaborated:

Most of my clients are newcomers. They do not know the system [and] the difference between a primary healthcare provider who is the GP [general practitioner] and an HIV specialist. If you have a primary healthcare provider who is vast in other medical areas than HIV, they can pick different things. Some [clients] may just see an HIV specialist who might just look at HIV, but they do not explore other things. Most HIV specialists just think about CD4 count, is your viral load undetectable, and that is it! Most clients that I see do not even have the information to know how to advocate for themselves, or they might have, but there is that power dynamics of feeling that this is a doctor and me I am just a patient, especially if the doctor is white.

A critical aspect of managing HIV and comorbidities is scheduling multiple medical appointments and accessing consistent treatment. Black participants detailed that scheduling multiple medical appointments and accessing clinical care for the various underlying health conditions they were living with was challenging and frustrating because of difficulty finding and booking appointments with healthcare providers, long wait times and financial instability. Financial instability also constrained their ability to afford transportation. Medical appointments such as clinical and follow-up visits, diagnostic and laboratory work, and social support services conflicted and competed with personal priorities and commitments such as childcare, school, food, and immigration processes. Mary elaborated: “sometimes you have a lot of different appointments; HIV or eye doctor’s appointment, and sometimes you have like 5 or 6 appointments in one month. It is not easy to attend all, and you need transport. I try to manage, but sometimes it is challenging.” Participants often missed HIV clinical care and follow-up medical visits because most appointments occurred during business hours. Catherine, who was living with multiple conditions, explained:

I miss HIV appointments because sometimes, I have medical appointments with two doctors. Maybe I have a diabetes appointment, and I have HIV appointments. I cannot make both because maybe they came at the same time. So, I have missed and cancelled several appointments.

#### Homelessness, housing instability, and the work of adhering to ART

Black participants in this study experienced homelessness (*n* = 7) and housing instability (*n* = 13). Those experiencing housing instability lived in emergency shelters, were precariously housed in shared accommodations, assisted living or transitional housings. Black people associated their precarious housing situations to financial instability, high rental costs, unemployment, and precarious immigration status. Homelessness and housing instability constrained ART adherence. Precariously housed participants feared that routinely taking medication in the presence of roommates and other shelter residents could lead to involuntary disclosure of their HIV status and subject them to stigma, discrimination, and violence. At the time of the interview, all the Black people we interviewed reported that they relied on Ontario Disability Support Program (ODSP) to access HIV medication and extended healthcare benefits, including housing, transport, special diet, dental, and eye care. The ODSP is a program under the Ontario Ministry of Community and Social Services (MCSS) that provides “longer-term income support for people with disabilities” [65]. John, who was on ODSP and living in a shared accommodated, articulated the impact of housing instability on adherence to ART:

We were only given about six hundred [CAD 600]. We could not use more than $600 [on rent]. So, the only option was for the four of us to share accommodation. We have to share the master bedroom with two people. The other roommates are in two smaller rooms. What this means is that these people don’t know my status [HIV]. I cannot put my meds anywhere. My meds go in my laptop bag. I cannot take them openly when my alarm goes on at 8 am. I have to take my meds in the bathroom. If my roommate is in the bathroom, I will wait for him to come out. Sometimes I forget. I have an alarm that reminds me that I need to take my medication at 8 am and then another one at 9 am. However, if I miss taking it and I go to work, you see that dynamic?

Black participants (*n* = 6) living in shelters reported that strict shelter regulations and practices made it hard to adhere to ART. Emergency shelter residents were required to vacate night-time shelters early in the morning every day and roam around until shelters reopen during evening hours. Such routines made it challenging for most participants to take medications as prescribed. Client support and accountability policies that organize the work practices of caseworkers produced perverse effects. Shelter rules requiring residents to report their daily itineraries to caseworkers every morning interfered with their medical appointments’ schedules. Additionally, caseworkers whose roles included risk assessment and monitoring their clients’ activities were seen as insensitive, having little compassion, and ignoring their clients’ privacy and confidentiality needs. Participants expressed that those caseworkers forced them into involuntarily disclosing their health conditions and reasons for medical visits. Fear of breaking shelter regulation and losing accommodation forced participants who had early morning appointments that required them to leave the shelter before their caseworkers reported to work to miss their clinical visit.

Black people and healthcare providers reported that homelessness and housing instability experiences intersected with social factors such as mental health, drug use, addictions, and violence, constraining their efforts to adhere to ART. Joyce, who was a frontline worker living with HIV, explained: “If you are homeless and on drugs or got mental health issues, you might not be able to take your medication the way that you are supposed to be taking your medication.” Healthcare providers acknowledged that housing was a significant barrier to ART adherence. They noted that high rental costs, unsafe living conditions, stigma, and discrimination compromised Black people’s adherence to ART and subjected Black people to violence, mental health, and addiction issues. Providers noted that Black patients feared having ART in their possession because of HIV stigma. A physician based at an HIV clinic elaborated:

Housing is a huge barrier. I hear from many people [that] housing in Toronto is a nightmare because of the high rent cost. It is horrific right now that many people have shared space. You will hear many people all living in one bedroom. It’s a lot of shared communal living. Here is the problem. People who live in shared living situations cannot have their pills stored inside medicine cabinets or anywhere communal. People often tell me that a roommate or a family stumbled upon their medication, realized they were HIV positive and kicked them out of the house. So, people will hide their medication or taking medication becomes secondary to their disease. Because of stigma, people are so protective that they do not want to access medication anymore.

#### Food insecurity, and the work of eating healthy and taking ART as prescribed

Black people reported that food insecurity constrained their efforts to adhere to ART and medical recommendations. Healthcare providers advise people living with HIV, and especially those with other medical conditions such as diabetes or hypertension to eat certain foods to stay healthy. Participants (*n* = 11) were prescribed ART that must be taken with food. Black people could not access or afford healthier and culturally responsive food options necessary to maintain a healthy lifestyle and accommodate their health conditions, dietary restrictions and ART prescription requirement because of inadequate income and competing priorities. Participants on ART prescribed to be taken with food reported missing their dosages because of lack of food. Catherine expressed:

One of the setbacks for African and Caribbean people adhering to their medication is that some medication works better if they take it with food and water. However, many people from Africa Caribbean backgrounds are barely surviving or have meagre income. They cannot access food to help the body boost the immune system…Most of the time, the price of good quality food was outside the means of people of Caribbean and African descent.

Black participants established a relationship between food insecurity, financial instability, and housing instability. Participants living in shelters were provided with unhealthy and unbalanced dietary food options such as canned, frozen, or catered meals. Further, shelter regulations and living arrangements restricted cooking within the premises. Shelter residents had no choice but to eat meals provided in the shelter. Accessing adequate and high-quality food competed with other priorities for participants with families and children, including paying rent and transportation costs. Participants noted that paying rent for family housing often consumed the limited income that they received from ODSP. David expressed:

All the money they [ODSP] give you goes to the rent. So, I cannot eat. I am on medication, and I have to take care of my kids. I know they [ODSP] give me money, but the money goes to the rent, so it’s like they do not give you anything. When they give you the money, and it all goes to the rent, that means you cannot eat, and the children cannot eat. If I can’t eat and I am told I need to eat before medication, where is the food? All the money I used to pay rent.

Participants who were prescribed ART that had to be taken with food and lived in shared accommodation or shelters reported that they took their medication without food because of privacy and confidentiality concerns and fear of stigma.

#### Financial instability, competing priorities and reliance on social welfare benefits

Lack of comprehensive public healthcare and treatment coverage or private insurance intersected with financial barriers, making it challenging for Black people living with HIV to access treatment for comorbidities and related healthcare services, and have optimal health outcomes. Black participants who were primarily unemployed or doing informal low-wage jobs expressed that they did not have drug coverage and could not pay out of pocket for healthcare services, including transportation costs, laboratory services, and medication. Participants noted that doctors often recommended and made referral for free healthcare services or elective lifestyle activities such as massage, gym, and healthy eating as alternative approaches to addressing health system barriers and accessing healthcare services that Black people needed but could not access. However, places offering free medical, support and elective services had long waiting lists and wait times. Physicians also cited challenges in referring patients to free health and wellness services. A physician elaborated:

You will often find that we do referrals for people to see a psychiatrist, and it takes two months. You find out two months later that nobody has called the patient. So, there is a problem in just getting patients seen, and there is even a bigger problem in getting the services that are not covered by OHIP, like counselling services. I would send a referral to the trauma program, and it is always rejected that “we are not taking any new patients.” So, it sounds good on paper, but it does not work in real life.

Many participants reported that the income benefits received from ODSP were limited and did not meet all their basic needs. It was challenging and stressful for participants with large families to afford housing, food, and recreational activities. David, who had a large family, was living with multiple comorbidities and was depending on ODSP for rent and food expressed:

That one [housing] is always giving me stress, constantly worrying. We are four; one boy, one girl, my wife and me. When I was alone, they paid rent 550 to 600 [CAD]. When my family came, they gave us [CAD] 918 for the rent. I cannot rent a two-bedroom apartment for 918, and I am sick. I am not working now.

Many participants indicated they wanted gainful employment to generate income to supplement inadequate ODSP benefits. However, strict policies and institutional practices regulating access to social services undermined their agency. Participants expressed tension between seeking gainful employment and continuing to depend on ODSP financially.

The ODSP policy states that its aim is “to help the province’s most vulnerable citizens” while working to “promote an ethic of self-reliance through employment” (MCSS, 2016). However, the process of establishing and maintaining eligibility for benefits subjected applicants or recipients of ODSP to punitive income and financial security assessments. Recipients of ODSP are required to report their income for assessment. Under Section 5 [1](c) of the ODSP Act, the asset limit is set at $40,000 for a single person, $50,000 for a couple and $500 for each dependent other than a spouse. All interest earned on assets within this ceiling is exempt from income under ODSP [66].

Black participants expressed feeling trapped within the welfare system because of these draconian and punitive policies and institutional practices. Noting that their health and survival were dependent on guaranteed access to ART and extended social benefits, participants expressed fear and worry over the possibility of losing their ODSP eligibility as a result of getting gainful employment. Participants feared they would be forced to channel all income made to paying for medication and have nothing left to cover basic needs like food, housing, and clothing. Indicating that Black people living with HIV are subjected to precarious employment conditions, minimum wage, and premature dismissal because of their lack of or limited education, anti-Black racism and disability, participants expressed that they could not afford medication and other basic needs. Thus, the need to continue accessing drug benefits becomes the primary justification for remaining on ODSP. Lucy, who expressed her desire to work but feared losing ODSP, articulated:

When I decide to remain on ODSP, I know the drugs will be expensive. If you are sure of getting the drug every month, your health is guaranteed. You are sure you will live well. You are not paying for it [ART]. I might be working every month. I think I’m strong [and] money is coming. What if I am unable to work? What happens? Will I be able to pay for my medication? I might not use what I have to pay rent. I do not know if I will even be able to save enough to pay rent.

Participants who decided to seek employment rather than remain on ODSP expressed that they had to discontinue ART because they could not afford to pay for treatment out of pocket. Doing informal jobs and working odd hours also contributed to participants’ non-adherence and inability to stay in care. For these reasons, many participants called for universal access to drug coverage, irrespective of their employment status. A participant stated: “that is why I said that medication should be there for everybody. The medication [insurance] should be 100% sure [universal] that you are getting your medication every month, even when working.”

#### The work of dealing with HIV related stigma, racial discrimination, and anti-black racism

Key informants and healthcare providers commonly noted that racial discrimination, systemic and anti-Black racism, prejudice, and stigma resulting from discriminatory and punitive policies produced inequities in accessing social determinants of health including gainful employment and healthcare, and subjected them to poverty, financial instability, and fear of losing healthcare benefits, leading to continued dependency on ODSP and feeling of being trapped in the welfare system. Participants expressed that HIV status and history of living on social welfare benefits added another layer of stigma and discrimination, denying them an opportunity to access gainful employment. Esther elaborated:

You leave ODSP and look for a job. Somehow in the paperwork, they are going to find out that you have HIV. Questions like, “what have you been doing in the last three months?” “I was on disability.” “Can you tell us what form of disability you have so that we can accommodate you?” They will not tell you why exactly they want to know, but they will tell you, “So that we can accommodate you.” Once you have been honest and sincere with them, the company’s accountant has to key in the insurance costs. You become gullible because the drugs are expensive.

Black participants living with HIV (*n* = 9) recounted how they were increasingly policed and surveilled within healthcare settings because of being Black and the perception that they were dangerous, aggressive, and a public threat. Charles, who was dealing with mental health issues at the time of the interview described his experiences within a mental health institution:

I started having mental health issues around the same time when I was diagnosed with HIV. It’s been difficult for me to access healthcare. If I am going through an anxiety episode, I feel that as a person of colour, I may be looked at as maybe aggressive, rude, or dangerous once I enter the healthcare system. I get sort of feedback based on how people react around me. You are sitting down and looking up and seeing like three or four security guards suddenly sitting there. I feel that it has to do with the colour of my skin. While the alarm [surveillance and policing] is this high, for a [person] with mental health issues like me, in my head, I start asking, why are all these happening? Is it just me? What is going on here?

Black participants and healthcare providers indicated these oppressive and violent experiences of stigma, racial discrimination and anti-Black racism negatively impacted Black people’s mental health, attitude, and behaviours towards seeking healthcare, and decision to adhere to ART. Black participants with precarious immigration status (*n* = 11) reported that they avoided, delayed, or stopped accessing healthcare because of fear that their HIV-positive status could be flagged in the immigration process and risk losing their immigration status, or face deportation or criminal charges. Uncertainties and confusion around when, where, and how to disclose one’s HIV status to healthcare professionals resulted in Black people experiencing prejudice, stigma, and threats of criminal charges. Rose explained how a dentist threatened her son and herself with a lawsuit for failing to disclose their HIV status:

They [dental staff] are not supposed to discriminate against us. They [staff] were wrong at first because they did not give us the form to fill before attending to us, and they have no right to do that. We fear it affecting our permanent residence application because we just got into the country. We do not want any issue with the police [or] with the courts. You know the fear is there. We want to be law-abiding citizens, and all our claims are accepted, and we get our permanent residence.

A frontline worker who self-identified as a Black person living with HIV expressed: “there is still HIV stigma in Canada. The challenge of stigma [is that] when someone is referred somewhere else, the way they are treated at the first appointment will determine whether they are retained in care or not.” A physician explained: “A woman from a Caribbean country was not taking her medication regularly… It is quite apparent that it is because of the stigma of HIV.

These violent and punitive experiences of racial discrimination, stigma and anti-Black racism made it difficult for Black people to adequately adhere to ART and reach an undetectable viral load, leading to poor health.

#### HIV criminalization, privacy concerns, mistrust, and the patient-provider relationship

Black people reported that they did not discuss their sexual health practice, risk, and needs with their healthcare providers due to ongoing HIV criminalization. In Canada, the criminal law is currently used to charge and prosecute people living with HIV who allegedly expose their sexual partners to HIV, fail to disclose known HIV-positive status before consensual sexual contact, or transmit HIV sexually [67, 68]. The Supreme Court of Canada (SCC) ruling in the cases of R. v. Cuerrier in 1998 and R. v. Mabior and R. v. D. C in 2012 set precedents where people living with HIV are legally required to disclose their HIV-positive status to sexual partners before engaging in sexual activities that pose what the Court calls a “realistic possibility of transmission” [69]. In 2018, the Attorney General of Canada issued a directive under section 10 [2] of the *Director of Public Prosecution Act* [70] directing that the Director of Public Prosecutions:

Shall not prosecute where a person living with HIV has maintained a suppressed viral load (i.e. under 200 copies of the virus per millilitre of blood) because there is no realistic possibility of transmission; shall not prosecute where the person has not maintained a suppressed viral load but used condoms or engaged only in oral sex or was taking treatment as prescribed unless other risk factors are present, because there is likely no realistic possibility of transmission in such cases; and shall prosecute using non-sexual criminal offences instead of sexual offences where this would better align with the individual’s situation, such as cases where the individual’s conduct was less blameworthy; and must take into account whether a person living with HIV has sought or received services from public health authorities, to determine whether it is in the public interest to pursue criminal charges.

Black people highlighted their vulnerability and fear of exposure to HIV non-disclosure criminal charges. Participants were concerned about the uncertainty of reaching and maintaining undetectability due to the above-discussed structural inequities that constrained their retention in care and adherence to ART. This uncertainty produced fear and negatively influenced Black people’s attitude towards healthcare systems and services. Black people also reported a lack of engagement in care and poor ART adherence due to concerns that accessing healthcare services would lead to the flaging of their HIV status within the immigration system, leading to the impairment of legal status and deportation applications. Fred articulated:

The law says you cannot be criminalized if you have an undetectable HIV viral load. However, there is already a caveat there. What if somebody decided you are detectable? What if all these other circumstances and life struggles make the viral load spark up? It can spike up. When you are sick or when you are unwell. So, when I have HIV, I am still a criminal. The law does not say I am not. So, when providing sexual history, I do not want to tell them. Some want to ask you about sexuality, and I do not want to tell them right because there are all these things. I still have an immigration case going on. I do not know who will have access to medical information. Already the refugee board has a lot of my medical information.

## Discussion

This study used institutional ethnography to explicate how structural violence embedded within social institutions and policies constrain retention in care, adherence to ART, and the ability of Black people living with HIV to reach undetectability. Mendenhall and colleagues state that “how we think about disease pathologies affects how we design policies and deliver care to those most affected by social and economic inequities” [71]. This study findings revealed that biomedical discourses and practices of HIV healthcare and treatment are extremely problematic for Black people living with HIV. Biomedical discourses of HIV healthcare and treatment and related policies and institutional practices, which focus on individual attitudes and behaviours ignore structural violence and inequities in determinants shaping the day-to-day social world of Black people living with HIV [3–5, 72, 73]. The distinction between a “undetectable” or “detectable” patient is often based on the degree to which a patient adheres to the perceived best practices of HIV healthcare and treatment. From a biomedical angle, people living with HIV are expected to access and remain engaged in care and consistently adhere to ART to achieve and maintain undetectability [17, 74–76]. However, from the standpoint of Black people encountering structural violence, achieving, and maintaining undetectable HIV viral load is more than a biometric marker attributed to successful ART treatment and adherence to healthcare. Structural violence which manifests through structural and social determinants inequities intersect to impede Black people’s ability to successfully engage in healthcare and adhere to ART [1–3, 12, 72, 77–80]. As a result, achieving and maintaining undetectability and optimal health outcomes remain a distant reality for Black people living with HIV. Therefore, dominant and objectifying biomedical discourses and practices of HIV healthcare and treatment which produce categories of “good” and “bad” patient position ACB people as deviant, irresponsible, and viral underclass, and reinforce past patterns of exclusion and pathologization of Black bodies.

This study established that structural violence embedded within social structures such as laws, policies and institutional practices, and manifest through systemic inequities constrain Black people’s retention in care and ART adherence, and ability to achieve and maintain undetectability, optimal health outcomes, and quality of life [14, 81], leading to structural violation of their human rights [37]. The manifestation of structural violence and violation of the human rights of Black people materialize from how laws and policy frameworks regulating public systems constrain Black people’s agency to the extent that they cannot equitably access health determinants. Article 12 [1] of the International Covenant on Economic, Social and Cultural Rights (ICESCR) recognizes “the right of everyone to the enjoyment of the highest attainable standard of physical and mental health” [82]. Although Canada ratified the ICESCR and the Canada Health Act (1982) commits under section 3 to “protect, promote, and restore the physical and mental wellbeing of residents of Canada and facilitates reasonable access to health services without financial or other barriers,” Canada does not include economic, social, and cultural rights like health in the Charter.

Food insecurity emerged as a significant barrier to ART adherence for Black people in this study. Food insecurity occurs “whenever the availability of nutritionally adequate and safe foods or the ability to acquire acceptable foods in socially acceptable ways is limited or uncertain” [83]. Food insecurity among Black people is shaped by race, poverty, unemployment, precarious immigration status, education, household composition, disability, competing priorities such as children, racial discrimination, and systemic and anti-Black racism embedded within socio-economic systems including labour markets, housing, and education. Prioritization of competing needs such as providing food for family members, securing stable housing, and paying for transportation and childcare due to financial instability limit Black people’s access to adequate, high-quality, and nutritious meals required for their health needs, dietary restrictions and ART adherence [84]. Studies have shown that food insecurity and poor ART adherence impact the pharmacokinetics, effectiveness, and efficacy of treatment, resulting in inadequate viral suppression, viral rebound, increased incidences of comorbidities, and suboptimal health outcomes [85–89]. These findings demonstrate that severe food insecurity is a human right and a public health issue for people living with HIV.

Homelessness and housing instability for Black people which is compounded by structural violence resulting from the unjust and discriminatory labour market and housing policies, and manifested through systemic and anti-Black racism impact retention in care, ART adherence, and achieving undetectability for Black people [81]. Precarious housing conditions produce privacy and confidentiality concerns, and fear of involuntary disclosure of HIV status, stigma and discrimination, loss of accommodation and violence. As a result, Black people living in crowded environments are forced to be non-adherent and non-compliant to treatment and prescription guidelines such as taking medication in a timely and consistent manner, and with food. Further, homelessness and housing instability produce and exacerbate violence, mental health conditions, drug use and addiction, leading to negative attitudes towards retention in care and ART adherence [90]. These findings contribute to a growing body of literature capturing the effects of housing instability on retention in care, ART adherence, health outcomes, and the quality of life of people living with HIV [91–93].

Black people living with HIV experienced an array of comorbidities, including high blood pressure, diabetes, gastric conditions, cardiovascular diseases, and elevated mental health conditions. Comorbidities were compounded by structural inequities, including stigma, racial discrimination, anti-Black racism, poverty, housing instability, unemployment, financial instability, punitive and unjust policies, and healthcare practices. Research examining synergistic epidemics or syndemics has illuminated how structural factors produce and perpetuate structural vulnerabilities, resulting in increased incidences of comorbidities and burden of HIV, and worsening clinical outcomes among people living with HIV [94, 95]. Drug use and addiction, history of war, violence, political conflicts, and pre-and post-immigration trajectories are associated with exacerbated mental health conditions, including depression, and post-traumatic disorder. Therefore, the syndemic interactions of comorbidities, adverse effects of social and structural inequities including racial discrimination, stigma and systemic and anti-Black racism, and inequitable access to SDH constrain Black people’s retention efforts in care, ART adherence, and reaching undetectability, leading to poor health outcomes and a violation of their right to health [96].

Time emerged as a structural determinant of health. Time shaped and constrained the health work process of retention in healthcare and adherence to ART and the overall health of ACB people. The time ACB people spent seeking healthcare services and the time medical professionals allocate to healthcare delivery relative to time each individual need to receive quality care were barriers to retention in care. Cumulatively, long wait times result from the time patients spent finding primary healthcare providers and specialists, booking appointments, processing referrals, and making clinical visits. The long wait and short consultation times are associated with the fee-for-service payment model and policy where healthcare providers bill each time a patient visits or revisits [97]. The fee-for-service model represents a competing interest because the financial scheme disincentivizes addressing multiple issues at once, and instead encourages physicians to allocate shorter consultation time and address one issue per visit to treat more patients per day and have higher-frequency unifocal visits [97]. Long wait times and short consultation time resulting from the fee-for-service and one issue per visit policies deny patients the opportunity to discuss all their health issues, leading to poor quality of service including untimely and inadequate diagnose of conditions. Additionally, these inequitable policies and institutional practices constrain patient-provider communication and relationships and diminish patient healthcare satisfaction. As a result, inadequate time in healthcare delivery harms Black people’s attitudes and decisions to consistently engage in healthcare, resulting in poor retention in care and ART adherence. Similar findings have been found in other studies [98–100]. Moreover, HIV clinical guidelines where follow-up visits are set at six-month to one-year intervals deny patients the opportunity to consistently seek treatment for evolving health conditions. Retention in care is also impacted by competing priorities such as work, school, childcare, job seeking, and immigration system engagements that conflict with medical appointments.

Prescription drugs are a critical component of HIV healthcare and response. Despite progressive scientific advancements in HIV response, lack of access to ART remains a major barrier to healthcare retention and adherence to ART for ACB people living with HIV. Canada is the only country in the world with a universal health care system that does not include universal coverage for prescription drugs [101]. Instead, Canada has an inequitable, inefficient and unsustainable patchwork system of private and public drug insurance coverage [102]. Private insurance plans are offered by employers and public insurance plans are provided by provinces and territories, all with a variety of premiums, co-payments, deductibles, and annual limits. The patchwork system leaves ACB people unable to afford and adhere to ART. Black people are unable to cover costs of ART through private insurance from employers or out-of-pocket because of financial barriers associated with poverty, unemployment, precarious immigration status, limited education and disability. The inherent need to have guaranteed access to ART force Black people to depend on social welfare benefits to access drug coverage and extended services such as housing, transportation, and special diet allowance. However, social welfare benefits remain inadequate and unable to meet the basic human needs of Black people living with HIV.

Findings show that ACB participants who were all on social welfare assistance programs including ODSP at the time of the study wanted to engage in gainful employment to supplement the limited social benefits, escape poverty, have adequate access to social determinants of health and achieve optimal quality of life. Despite the inadequacy of social welfare benefits and readiness of welfare recipients to work, strict welfare policies and institutional practices regulating eligibility for social welfare benefits constrain Black people’s integration into the labour market, leaving them trapped within the system. According to social welfare legislative policies and directives, social welfare beneficiaries who fail to declare their income risk losing their benefits and being criminalized [103]. The organization of social welfare programs where recipients seeking gainful employment are faced with the impossible choice of either remaining in the welfare system or risk losing benefits, subject Black people to what other scholars refer to as the ‘perverse incentives’ [103, 104]. We argue that Canada’s social welfare system and related policies result in perverse incentives that produce and perpetuate structural violence. The perverse incentive for social welfare beneficiaries to remain on welfare programs in order to access sustained health benefits including drug coverage and housing allowance constrain ACB people’s self determination to be self-sufficient through gainful employment while adhering to HIV healthcare practices. Because of the social welfare system’s structure, policies and perverse incentives, Black people living with HIV in Canada are trapped within the social welfare system with no gainful employment, creating a cycle of intergenerational poverty. Furthermore, Black people on social welfare are forced to work under precarious conditions including working ‘under the table’ to retain access to benefits and supplement limited social benefits. Moreover, the adverse effects of perverse incentives and structural violence force some ACB people to drop out of care and stop taking their ART. These findings complement studies that have looked at the impact of social welfare benefits such as ODSP on people with disabilities [105].

Punitive and exclusionary law, policies, and institutional practices at the intersection of healthcare, public health, immigration, criminal justice system that punish, charge, and criminalize people living with HIV, including for non-disclosure of their positive status and failure to reach or sustain undetectable viral load level adversely impact retention in care and ART adherence. New prosecutorial directives stating that the federal and Ontario governments will no longer be prosecuting people living with HIV who consistently maintain an undetectable viral load for a period of six months raise essential questions around equity and social justice, considering that HIV criminalization legal frameworks apply to people who are not engaged in care and have detectable viral loads [70]. The law, which uses viral undetectability as a legal benchmark for laying criminal charges, essentially ignores structural inequities shaping Black people’s social world, exposing them to risk of criminalization due to their race, poverty, detectable viral load, and HIV-positive status. As a result, the criminal law prosecuting people living with HIV for alleged HIV non-disclosure intersects with unjust immigration and healthcare policies to negatively impact Black people’s attitude towards healthcare providers and services [67]. Privacy concerns and provider mistrust, together with the fear of prosecution for alleged HIV non-disclosure, loss of immigration status, and deportation impact Black people’s willingness to access healthcare or share information about their sexuality and sexual health with healthcare providers. Furthermore, these violent and exclusionary policies and practices produce and exacerbate adverse mental health outcomes and negatively impact Black people’s attitudes and decisions to engage in HIV care and adhere to ART. These findings are consistent with results from several other studies that have looked at the impact of HIV criminalization laws on public health prevention practices and human rights [67, 106, 107]. Studies have shown that synergistic syndemic conditions resulting from structural violence trigger chronic psychological and physiologic stress response, which interferes with the effectiveness of ART ability to suppress one’s viral load [108, 109].

Biomedical discourses and evidence on ART and undetectability have informed the development of several national and global initiatives focusing on ending HIV/AIDS. The global 90–90-90 targets of the Joint United Nations Programme on HIV/AIDS (UNAIDS) aim to have 90% of all people living with HIV know their status, 90% of those diagnosed receive ART, with the third target focusing on having 90% of those on treatment achieve viral suppression by 2030 [110]. In 2016, the United Nations General Assembly of states developed the global Fast Track Initiative (FTI) to “fast-track” the HIV/AIDS response by removing structural barriers, expanding access to vital HIV services, and ending HIV transmission in cities with a high prevalence of HIV [111]. In 2019, using the FTI framework, the Toronto to Zero (TTZ) community-based initiative was started in collaboration with people living with HIV and other community members, community-based AIDS service organizations (ASOs), clinicians, public health agencies, researchers, and government stakeholders to fast track and successfully end the HIV epidemic in Toronto, Canada [112]. The TTZ aims to reduce the number of new HIV transmissions by two-thirds surpassing the UNAIDS 90–90-90 targets, achieve 95–95-97 and ensure all key populations benefit from care and treatment. The TTZ initiative added a “fourth 90” target that aims to improve the health of people living with HIV and end HIV stigma and discrimination [112].

Structural violence resulting from unjust welfare, housing, immigration, labour market and healthcare policies, and institutional practices subject Black people into permanent positions of homelessness, housing instability, food insecurity, inability to afford healthcare-related costs including transportation, and dependency on the social welfare system, violating their fundamental human rights [103]. The violation of human rights, particularly the right to health including equitable access to social determinants of health undermine national and global efforts to control and end the HIV/AIDS epidemic. As a result, Black people are left behind in the HIV response. This is despite the much-celebrated biomedical advances, the basing of Canada’s healthcare systems on equity and social justice, and the increasing recognition of access to quality and adequate healthcare as a human right. Results from our study have important implications for research, practice, and public policy reform. The findings illuminate the importance of an equity lens and rights-based approach to HIV healthcare, treatment and prevention among Black people living with HIV and other vulnerable populations in Canada and globally. Achieving undetectability and ending HIV is not an isolatable aspect limited to individual attitudes and behaviours but constitute a work process socially mediated by a host of structural factors and forces that operate to restrict the right to health including equitable access to healthcare and health determinants. Thus, the right to health directs governments to protect vulnerable and marginalized populations from discriminatory and violent structural policies that limit access to health determinants. World Health Organization’s (WHO) “Health in All policies” calls on healthcare workers to collaborate with state agents across various government institutions in advocating for, developing, and implementing policies, practices and interventions that address health inequities [113]. Implementing structural interventions that guarantee universal and comprehensive access to health determinants would promote conditions that significantly improve retention in care and adherence to ART, increase viral suppression, and lead to optimal health [93, 114]. This approach complements Ontario’s HIV/AIDS Strategy 2025, which notes that systemic changes must focus on the health of people living with HIV and the broader structural factors driving the epidemic, including socio-economic inequities, stigma and discrimination, and social determinants of health [115].

To end HIV/AIDS by 2030, “leaving no one behind,” governments must accelerate efforts to “achieve universal access to comprehensive HIV prevention programmes, treatment, care and support” [116, 117]. Implementation of a universal and comprehensive national public medication insurance program (phamacare) that provides drug coverage including out-patient prescription expenses would facilitate equity in healthcare including access to ART treatment. Furthermore, governments must eliminate punitive laws and policies that criminalize poverty, disability, and people living with HIV for HIV non-disclosure.

The harmful effects of structural violence on Black people’s lives call for integrating trauma-informed knowledge and care into healthcare programs, services, and policies. There is growing evidence that trauma-informed HIV healthcare interventions improve healthcare access and retention, ART adherence, viral load suppression, health outcomes, and quality of life [118–120]. The findings that limited access to healthcare providers with knowledge about HIV and lack of cultural competency result in suboptimal care and poor retention in care highlight the significant need for culturally relevant, safe and responsive tailored healthcare programmes and interventions that serve the specific needs of Black people [121, 122]. Studies show that interventions focusing on training healthcare providers on cultural competency improve providers’ understanding of culturally responsive healthcare and services and strengthen patient-provider relationships [123, 124].

Eliminating structural forms of violence and advancing social justice is timely, given the documented disproportionate impact of the COVID-19 pandemic on Black and racialized communities, particularly those living with HIV [125]. The syndemic of COVID-19, HIV, comorbidities, and racial inequities among Black communities foreground the need to centre social injustices including systemic and anti-Black racism in research. Further, the study findings justify the need for race-based data collection.

## Conclusion

Black communities in Ontario are structurally disadvantaged. Biomedical advances in HIV healthcare and prevention practices, policies, and programs that aim to achieve “zero” HIV transmission are not equitably benefiting Black communities. Structural violence ingrained in healthcare and health-related systems, legislative frameworks, policies, and institutional practices has produced inequities in accessing social determinants of health for Black communities living with HIV, leading to poor retention in care, ART adherence, health outcomes, and difficulty achieving and maintaining undetectability. These structural forms of violence result in a violation of Black people’s right to health. Therefore, the practices surrounding HIV healthcare and the concept of undetectability should not be conceptualized as an individual responsibility but rather as human rights and social justice issues. As such, turning the tide on the HIV epidemic can only happen if structural factors perpetuating violence among Black communities and other vulnerable populations are identified and eliminated. Understanding how structural violence is embedded in the social fabric of Canada and produce inequities allows for identifying forces that influence HIV healthcare practices and health outcomes beyond the local and individual levels. Hence, recognizing the structural barriers to retention in care, adherence to ART, and maintaining viral suppression as acts of structural violence is a pathway to advancing equitable policies and institutional practices that enhance HIV healthcare and prevention practices, leaving no one behind. Therefore, achieving global targets that aim to end HIV/AIDS by 2030 and improve health outcomes calls for reforming and eliminating laws, policies, and institutional practices that produce structural and social inequities, and reinforce structural violations of human rights to health.

## Data Availability

The dataset(s) supporting the conclusions of this article is (are) included within the article.
